# Synthesis optimisation and characterisation of chitosan-calcite adsorbent from fishery-food waste for phosphorus removal

**DOI:** 10.1007/s11356-019-07570-0

**Published:** 2020-01-11

**Authors:** Sabolc Pap, Caroline Kirk, Barbara Bremner, Maja Turk Sekulic, Stuart W. Gibb, Snezana Maletic, Mark A. Taggart

**Affiliations:** 1 Environmental Research Institute, University of the Highlands and Islands, Thurso, Caithness, Scotland KW14 7JD UK; 2 Faculty of Technical Sciences, Department of Environmental Engineering and Occupational Safety and Health, University of Novi Sad, Novi Sad, Serbia; 3 School of Chemistry, University of Edinburgh, David Brewster Rd, Edinburgh, EH9 3FJ UK; 4 Faculty of Science, Department of Chemistry, Biochemistry and Environmental Protection, University of Novi Sad, Trg Dositeja Obradovića 3, Novi Sad, Serbia

**Keywords:** Waste management, Deacetylation, Adsorption, Nutrient recovery, Wastewater treatment, Circular economy

## Abstract

Here, Box-Behnken design (BBD) approaches were utilised to optimise synthesis methodology for the chitosan-calcite rich adsorbent (CCM) made from fishery-food waste material (crab carapace), using low-temperature activation and potassium hydroxide (KOH). The effect of activation temperature, activation time and impregnation ratio was studied. The final adsorbent material was evaluated for its phosphorus (P) removal efficiency from liquid phase. Results showed that impregnation ratio was the most significant individual factor as this acted to increase surface deacetylation of the chitin (to chitosan) and increased the number of amine groups (–NH_2_) in the chitosan chain. P removal efficiency approached 75.89% (at initial P concentration of 20 mg/L) under optimised experimental conditions, i.e. where the impregnation ratio for KOH:carapace (g/g) was 1:1, the activation temperature was 105 °C and the activation time was 150 min. Predicted responses were in good agreement with the experimental data. Additionally, the pristine and CCM material were further analysed using scanning electron microscopy with energy dispersive X-ray spectroscopy (SEM/EDX), Brunauer-Emmett-Teller technique (BET), X-ray diffraction (XRD), Fourier transform infrared spectroscopy (FTIR) and thermal gravimetric analysis (TGA). Characterisation showed enhancements in surface chemistry (introducing positively charged amine groups), textural properties and thermal stability of the CCM.

**Electronic supplementary material:**

The online version of this article (10.1007/s11356-019-07570-0) contains supplementary material, which is available to authorized users.

## Introduction

Phosphorus (P) is an essential macro nutrient for plants and animals, but excessive dissolved orthophosphate ions in aquatic ecosystems can lead to a deterioration in habitat quality due to eutrophication (Mitrogiannis et al. 2017). Human activities that result in discharges of industrial, domestic and agricultural wastewater are often responsible for the enrichment of surface waters with P (Markou et al. 2015).

Various techniques have been used for P removal from P-rich effluents, including chemical precipitation and crystallisation (Huang et al. 2017), biological treatment (Yang et al. 2018), membrane technology (Furuya et al. 2017), constructed wetlands (Du et al. 2017), ion exchange (Bui et al. 2018) and adsorption (Yu et al. 2017). Physico-chemical methods to remove P during water treatment are either too expensive (i.e. membrane processes), whilst chemical precipitation is costly due to the need for additional metal salts (such as iron). Alternatively, whilst biological processes are low cost, removal efficiency is commonly < 30%; hence, additional P removal techniques may also be required (Alshameri et al. 2014). Further, biological processes and chemical precipitation are generally not suitable for P remediation at low concentrations (Lu and Liu 2010). P recovery (and potentially re-use) through adsorption has been identified as a promising research area since this may involve low energy consumption, provide various economic benefits, be simple and be highly effective even at low concentrations (Haddad et al. 2018; Pap et al. 2018).

To improve the competitiveness of any adsorbent in a commercial market, three key criteria need to be fulfilled: (a) low production cost, (b) high product yield and (c) high removal efficiency/adsorption capacity. In this case, if a P saturated adsorbent can also be used as a P-rich fertiliser in agriculture, this would also be highly consistent with the principles of a ‘circular economy’, whereby re-use or recycling of waste materials is optimised to extract maximum value (Zheng et al. 2019).

Recently, many newly developed adsorbents for P removal have been investigated, including biochars (Haddad et al. 2018), biomass (*Phragmites* sp.) (Markou et al. 2015), magnetite (Yu et al. 2017), zeolite (He et al. 2017), sepiolite (Yin et al. 2013), red mud (Ye et al. 2015), iron oxides (Ajmal et al. 2018) and mussel shells (Paradelo et al. 2016). Recovery of P from wastewater through adsorption onto natural solid waste material may provide an additional low-cost alternative, which could also create a P-rich product that could be re-purposed (i.e. as fertiliser), provided the final material is also low in other co-adsorbed contaminants (Dai et al. 2017). Crab carapace (*Cancer pagurus*), a common waste by-product from the fishery and seafood industry (i.e. generated in the millions of tons annually worldwide), is widely disposed of in landfill (Lu et al. 2007). Re-valorising and re-purposing such waste into a high value-added product may have multiple benefits.

Here, thermochemical activation using KOH was utilised to produce a more effective P adsorbent from raw waste crab carapace. For adsorbent preparation, a response surface design (RSD) tool showed to be a convincing statistical optimisation process. RSM determine a connection between effects and interactions and a group of variable parameters, leading to a better understanding of the minimum number of experimental runs needed to estimate the effect of those parameters and identify optimal processing conditions (Das and Mishra 2017; Montgomery 2017). A Box-Behnken design approach was further used to predict (a) the optimal impregnation ratio of KOH to crab carapace (g/g), (b) the ideal activation temperature and (c) the optimal time to prepare the adsorbent (the ultimate aim being high removal efficiency). The main aims of this work were to (i) thermochemically transform crab carapace into a valuable P adsorbent (with improved surface chemistry/textural properties), using an efficient activating agent and minimum activation time and temperature; (ii) investigate the influence of sorbent processing factors on the final properties of the adsorbent (using RSM) and (iii) highlight and discuss changes/improvements in the physico-chemical properties of the adsorbent, as prepared under optimal conditions. The novelty of this research lies in selection of the abundantly available fishery-seafood waste as precursor in low-temperature adsorbent activation to remove phosphate ions.

## Material and methods

### Adsorbent preparation

The raw source material for the chitosan-calcite–based adsorbent produced here was crab carapace from brown crab (*Cancer pagurus*). Carapace was collected (as food waste) from a seafood restaurant in Scrabster, Scotland.

During the initial experiment, the influence of chemical and thermal treatment was tested on P removal. For this, we first undertook (1) chemical modification with HCl (1 M), (2) chemical modification with KOH (1 M) and (3) thermal activation by heating at 600 °C for 2 h. The raw material was milled, washed (with tap water), dried at room temperature and then sieved to < 100 μm. The two chemical modifications were carried out using 10 g of crab carapace, by soaking in 100 mL of acid or base solution, whilst shaking at 150 rpm on a flat-bed shaker for 2 h at room temperature. Lastly, the three obtained materials were rinsed several times with Type I water (Milli-Q® Direct 8/16 System) to remove free chemicals/ions, and dried at 105 °C.

Based on the obtained results from initial experiments, KOH modification was chosen for further study/optimisation. Ten grams of sieved raw crab carapace was first mixed at different mass ratios with a KOH (at 0.10–1.00 g of KOH per gram of material) and the mixtures were placed into crucibles. These were placed into a muffle furnace and heated from room temperature up to different maximum/final temperatures (80–500 °C), which were then held for different times (30–180 min). After cooling, the resultant adsorbent samples were rinsed with Milli-Q several times to eliminate any by-product residues, then dried at 105 °C for 2 h and stored in glass bottles. The final optimised adsorbent is referred to here as CCM.

### Experimental design using response surface methodology

Optimal process settings and weak points of the experiment were identified using BBD—the aim being to maximise final P adsorption (*Y*). Justification and advantages of the BBD compared to other optimisation methods are previously discussed by Ferreira et al. (2007). The following preparation parameters were studied: (*X*_1_) impregnation ratio—IR, (*X*_2_) activation time and (*X*_3_) activation temperature. The low, mid and high levels of each variable were designated as − 1, 0 and + 1. These three levels, together with the respective ranges used, were chosen based on a literature review, ideal cost requirements and outcomes from preliminary experiments. Process parameters and their coded levels used are summarised in Table 1.

**Table 1:** Process parameters and their coded levels used for the BBD

Variables/parameters	Code	Units	Coded variable levels		
			− 1	0	+ 1
IR	*X*_1_	g/g	0.10	0.55	1.00
Activation time	*X*_2_	min	30	105	180
Activation temperature	*X*_3_	°C	80	290	500

A polynomial equation was employed to predict the optimal process point, expressed as Eq. (1):

1 $$ Y={\beta}_0+{\sum}_{i=1}^k{\beta}_i{X}_i+{\sum}_{i=1}^k{\beta}_{ii}{\left({X}_i\right)}^2+{\sum}_{i=1}^{k-1}{\sum}_{j=2}^k{\beta}_{ij}{X}_i{X}_j $$

where *β*_0_, *β*_i_, *β*_ii_ and *β*_ij_ are regression coefficients. *β*_0_ is a constant term which corresponds to the response when the value of *X*_i_ is zero for each parameter; *β*_i_ is the linear effect term; *β*_ii_ is the square effect term; and *β*_ij_ is the interaction effect term. *X*_i_ and *X*_j_ are the variables that represent the important parameters affecting the characteristic of the process being carried out. *k* is the number of variables. Minitab 18 (Minitab, Inc., USA) and SigmaPlot (Systat Software Inc., USA) were used to generate the statistical experimental design, analyse and represent the observed data (Gao et al. 2015).

### Material characterisation and analytical methods

The P stock solution (1000 mg/L) used was prepared by dissolving potassium dihydrogen phosphate (KH_2_PO_4_ - Sigma-Aldrich, UK) in Milli-Q water; desired working solutions were prepared by serial dilution of this stock. Potassium hydroxide (KOH - Sigma-Aldrich, UK) and other chemical reagents used were all analytical grade reagents.

The surface structure and elemental composition of raw/untreated crab carapace and the CCM adsorbent were determined using a scanning electron microscope (SEM; Topcon SM-300) equipped with a Titan XPP analogue X-ray pulse processor (EDX). Textural characterisation was determined using nitrogen adsorption at 77 K using an Autosorb iQ instrument (Quantachrome, USA). FTIR spectra (Perkin Elmer Spectrum) were collected over the range 500–4500 cm^−1^. X-ray diffraction (XRD) data were collected on a Bruker D2 Phaser diffractometer in reflection geometry, using Cu Kɑ over the two-theta range 6–60° for a total data collection time of 10 min. Samples were prepared as thin smears by grinding them in acetone and placing the resulting suspension on a silicon substrate. The thermal stability of the materials was determined by thermogravimetric analysis (TGA), and recorded using a thermo analyser (STA 449 C Jupiter – Netzsch).

P concentrations in solutions were measured using a SEAL AQ2 Discrete Analyser (Seal Analytical, UK) and the antimony-molybdate reaction with ascorbic acid as the reductant (LOD = 0.004 mg/L). The resultant blue colour is measured at a wavelength of 880 nm (APHA 2005). The instrument was calibrated within its linear range; correlation coefficients were always > 0.98; blanks and external standards were used to ensure quality control (QA/QC).

### Phosphorous adsorption experiments

In order to evaluate the impact of material preparation/optimisation conditions, on resultant adsorption performance, 200 mg of each CCM produced (17 samples using the various methods above) was suspended in a 50 mL P solution in an Erlenmeyer flask. Flasks were then stirred on an orbital shaker (IKA KS 260) at 150 rpm for 120 min at room temperature (22 ± 1 °C). Initial P concentrations of 20 mg/L were used. Suspensions were filtered through 0.45 μm PTFE (polytetrafluoroethylene) disposable syringe filtration membranes (Fisher Scientific), and the percentage of P removal, *R* (%), determined using the following equation Eq. (2):

2 $$ R\ \left(\%\right)=\frac{C_0-{C}_{\mathrm{e}}}{C_0}.100 $$

where *C*_0_ is the initial P concentration and *C*_e_ is the residual P concentration (mg/L). All adsorption experiments were run in triplicate. The sequential of the experimental setups is shown schematically in Fig. S1.

The P adsorption capacity was determined using Eq. (3):

3 $$ {q}_{\mathrm{e}}=\frac{\left({C}_0-{C}_{\mathrm{e}}\right)}{m}.V $$

where *q*_e_ is related to the amount of P adsorbed at equilibrium (mg/g), whilst *m* (g) and *V* (L) are the adsorbent dosage and volume of the solution used, respectively.

The selectivity of adsorbent was studied in 50 mL solutions containing 20 mg/L of P and 20 mg/L of each the following anions: NO_3_^−^, Cl^−^, CO_3_^2−^ and SO_4_^2−^ (total ion concentration of 100 mg/L).

## Results

### Influence of different thermochemical treatments on final P adsorption

**Fig. 1:**
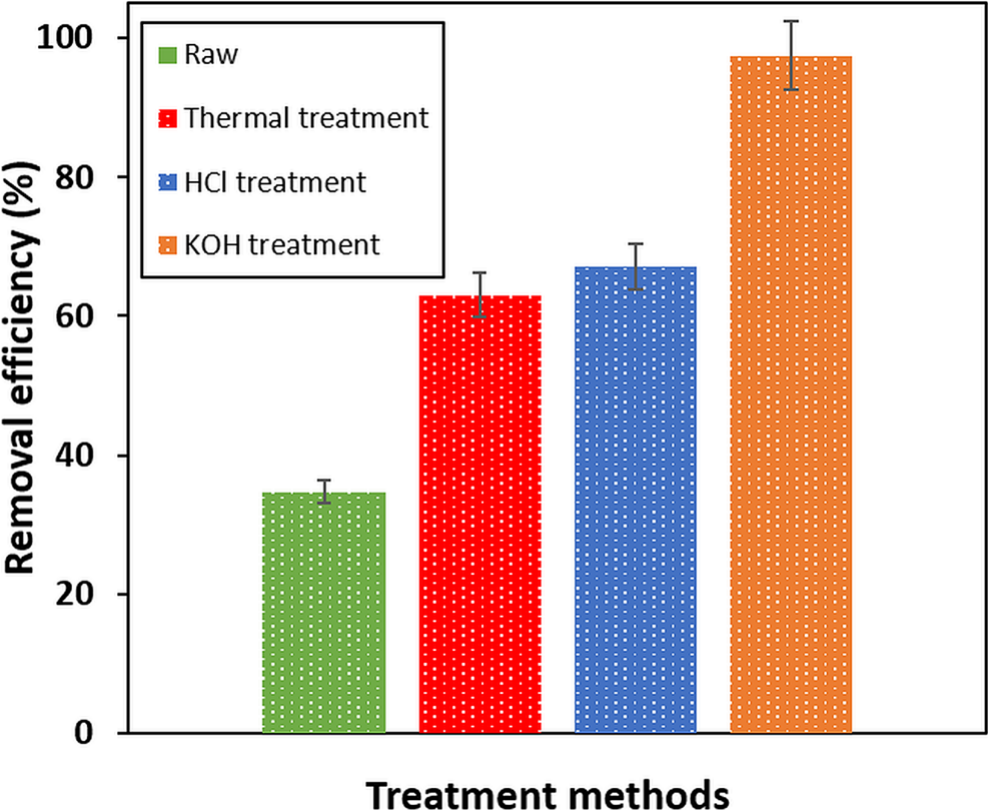
P removal efficiency of raw material and with different treatments used during the initial experiments (Note: thermal activation by heating at 600 °C for 2 h; chemical treatment with HCl (1 M); and chemical treatment with KOH (1 M); adsorbent dose: 200 mg, rotation speed: 150 rpm, contact time: 120 min, temperature 22 ± 1 °C and initial P concentrations of 20 mg/L)

Pristine, completely untreated crab carapace had a P removal efficiency of ~ 35% (Fig. 1). Likewise, when comparing adsorption results for materials undergoing thermal or chemical treatment (Fig. 1a), P removal efficiency increases from ~ 35 to ~ 63% after heating at 600 °C for 2 h. HCl treated (1 M HCl treatment for 2 h at room temperature) material gave similar results to the thermally treated sample. However, KOH activated adsorbent was much more efficient (1 M KOH treatment for 2 h at room temperature). The removal efficiency in this case increased from ~ 35 to ~ 97%. Based on these results, the KOH-treated material was then used for further optimisation.

### Box-Behnken design results

The number (N) of experiments required for the BBD model is defined as Eq. (4):

4 $$ N=2k\left(k-1\right)+{C}_0 $$

where *k* is the number of variables/factors and *C*_0_ is the number of central points. Given three factors (activation time, temperature and impregnation ratio), the BBD model consists of 12 trial runs and 5 central points. The central points were used to determine the experimental error and the reproducibility of the data.

The experimental data was obtained using the variables represented in Table 2. An independent quadratic model was used to fit the model with the experimental data. Table 2 gives the BBD matrix experimental results and the predicted P removal data. The final model is given in Eq. (5).

5 $$ Y=11.78+69.3\ {X}_1+96.0\ {X_1}^2-0.000275\ {X_2}^2+0.0813\ {X}_1{X}_2 $$

where *X*_1_ and *X*_2_ are the coded terms for the three variables selected. A positive sign in front of each term indicates a synergistic effect, whereas a negative sign indicates an antagonistic effect (Senthilkumar et al. 2017). Results were then analysed using analysis of variance (ANOVA) to assess the goodness of fit.

**Table 2:** Experimental design matrix and dependent variables attributed to the factors used for the BBD

Run no.	IR (g/g)		Activation time (min)		Activation temperature (°C)		Removal efficiency (%)	
	*X*_1_		*X*_2_		*X*_3_		*Y*_1_	
	Coded	Actual	Coded	Actual	Coded	Actual	Observed	Predicted
1	− 1	0.10	− 1	105	0	80	30.75	35.53
2	1	1.00	− 1	105	0	80	73.00	70.78
3	− 1	0.10	1	105	0	500	18.50	20.72
4	1	1.00	1	105	0	500	91.50	86.72
5	− 1	0.10	0	30	− 1	290	35.50	32.41
6	1	1.00	0	30	− 1	290	81.50	85.41
7	− 1	0.10	0	180	1	290	43.50	39.59
8	1	1.00	0	180	1	290	84.75	87.84
9	0	0.55	− 1	30	− 1	80	24.50	22.81
10	0	0.55	1	30	− 1	500	31.00	31.87
11	0	0.55	− 1	180	1	80	37.00	36.12
12	0	0.55	1	180	1	500	26.50	28.19
13	0	0.55	0	105	0	290	47.50	46.10
14	0	0.55	0	105	0	290	42.00	46.10
15	0	0.55	0	105	0	290	47.50	46.10
16	0	0.55	0	105	0	290	48.50	46.10
17	0	0.55	0	105	0	290	45.00	46.10

### ANOVA results

**Fig. 2:**
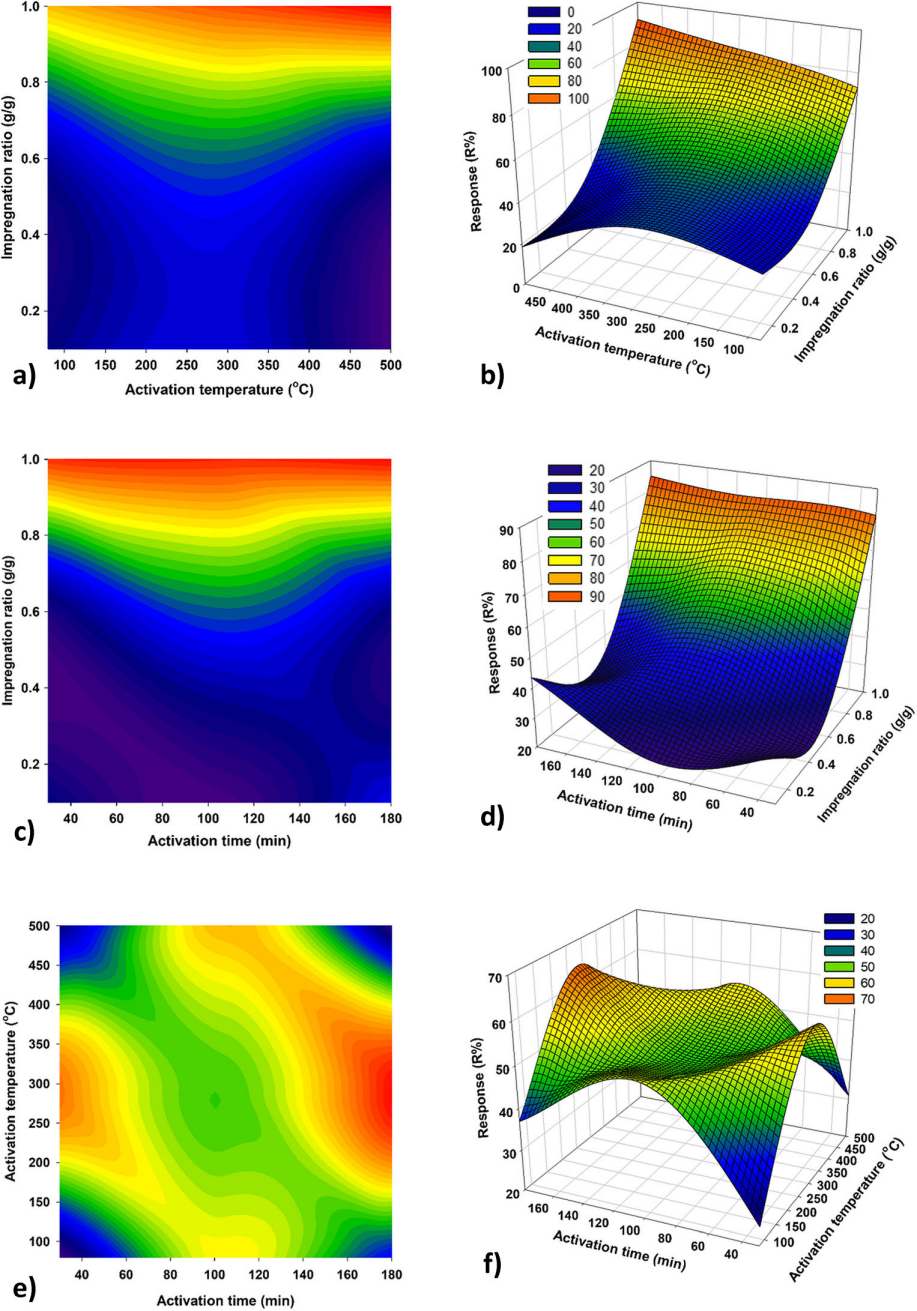
Contour and three-dimensional response surface plots regarding the effect of IR and activation time (**a** and **b**), IR and activation temperature (**c** and **d**) and activation temperature and activation time (**e** and **f**) on phosphorus adsorption

Table 3 shows the ANOVA analysis. Significant model terms for P removal were *X*_1_, *X*_1_^2^, *X*_2_^2^, and *X*_1_*X*_2_ (*p* < 0.05). The linear term of IR (*X*_1_) had the greatest effect on P removal, followed by the quadratic terms for IR and activation time (*X*_1_^2^ and *X*_2_^2^) and the interaction term between IR and activation time (*X*_1_*X*_2_). Figure 2a–d shows that for the conditions studied, removal of P increased with increasing IR. Figure 2e–f shows that the interaction between activation temperature and time has little/no significant influence on P adsorption. However, Fig. 2a–d demonstrates that a longer activation time and a moderate activation temperature (in combination with IR) slightly enhance P adsorption efficiency. At an IR of 1.0, the greatest P adsorption was achieved.

**Table 3:** ANOVA results for the response surface quadratic model for P removal efficiency

Source	Sum of squares	Degree of freedom	Mean square	*F* value	*p*
Model	7661.16	9	851.24	42.52	0.000^s^
*X*_1_	5125.78	1	5125.78	256.01	0.000^s^
*X*_2_	0.63	1	0.63	0.03	0.864^n^
*X*_3_	46.32	1	46.32	2.31	0.172^n^
*X*_1_^2^	1592.85	1	1592.85	79.56	0.000^s^
*X*_2_^2^	617.74	1	617.74	30.85	0.001^s^
*X*_3_^2^	75.61	1	75.61	3.78	0.093^n^
*X*_1_*X*_2_	236.39	1	236.39	11.81	0.011^s^
*X*_1_*X*_3_	5.64	1	5.64	0.28	0.612^n^
*X*_2_*X*_3_	72.25	1	72.25	3.61	0.099^n^
Residual	140.15	7	20.02	–	–
Lack of fit	112.45	3	37.48	5.41	0.068
Pure error	27.70	4	6.93	–	–
Cor total	7801.32	16	–	–	–

^s^Significant at *p* < 0.05

^n^Not significant at *p* > 0.05

High *R*^2^ value of 0.982 ensured the suitability of the quadratic models to the experimental data. Additionally, the ‘Lack of fit *F* value’ of 5.41 suggests the ‘Lack of Fit’ was not significant relative to the pure error, which indicated that the model was significant. Fig. S2 indicates the relationship between predicted and experimental values of P removal. The adjusted determination coefficient (adj. *R*^2^) was 0.959. This implies that the models could represent 95.9% of observed variability for P removal response. The ‘Pred. *R*^2^’ of 0.764 was in reasonable agreement with the ‘adj. *R*^2^’ of 0.959 because the difference between them was within 0.2 (Yuan et al. 2018).

### Optimisation, verification, adsorption capacity and selectivity

The best possible outcome (best P removal efficiency) was determined by numerical optimisation of the overall desirability function. To achieve maximum desirability and reduce preparation costs (keeping the electricity usage low), the activation temperature was set between 80 and 105 °C. Activation temperature has the greatest impact on production cost, but had the low impact on P removal efficiency (18.5%). Other parameters were set as per the optimisation process. Within the set temperature range, the predicted optimal values were therefore as follows: *X*_1_ = 1.0 g/g, *X*_2_ = 105 °C, and *X*_3_ = 150 min, to achieve maximum P removal of 75.89%, with an overall desirability result of 0.964. The verification was then examined by running experiments using these conditions, and the removal efficiency obtained in this experiment was 78.11%. Table S1 shows the predicted and experimental values at these optimum conditions.

The influence of initial P concentration on P adsorption was investigated within a concentration range from 1.0 to 50 mg P/L, to obtain the maximum adsorption capacity. As shown in Fig. S3a and S3b, P adsorption capacity gradually increased as the initial P concentration increased. Also, rapid adsorption was observed in the first 5 min, and then, the process slowed down. The system reached equilibrium after ~ 120 min. Maximum adsorption capacities was 21.56 mg P/g at 22 °C, which was superior to that found in many other literature using similar materials (Chen et al. 2012; Park et al. 2018). As shown in Fig. S3c, in solution with coexisting anions, variable effects on P removal were noted (the negative influence of carbonate was most significant, causing ~ 30% reduction in adsorption). In mixtures where all these anions were present, P removal efficiency decreased from ~ 90 to ~ 45%. It must be emphasised, that further adsorption studies will be conducted with real final effluents to test selectivity of the proposed adsorbent.

### Characterisation of the raw and optimised CCM adsorbent

#### SEM-EDX

**Fig. 3:**
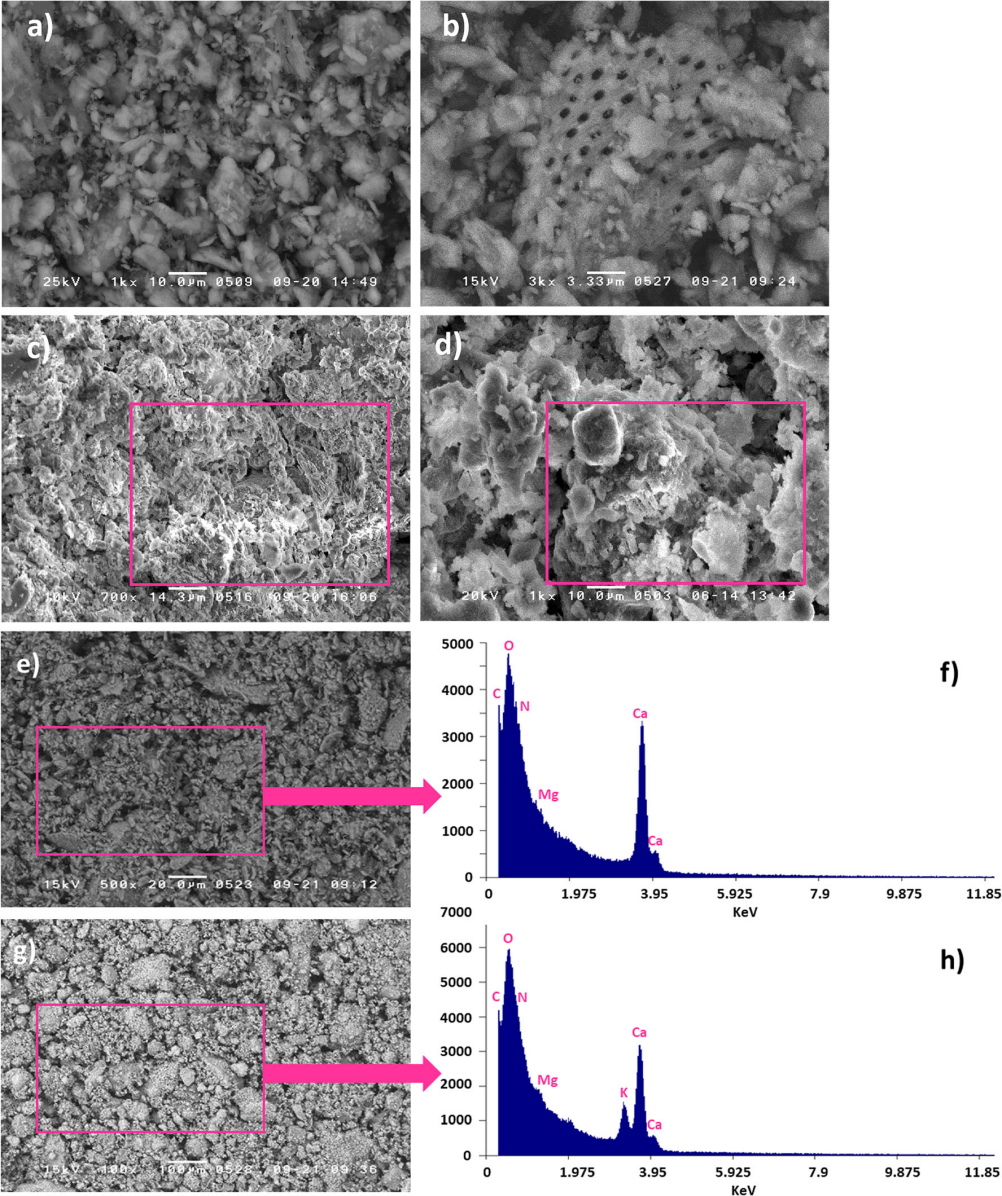
SEM micrographs of **a**–**b** pristine crab carapace (bar lengths 10 μm and 3.33 μm; magnification × 1000 and × 3000) and **c**–**d** optimised CCM adsorbent (bar lengths 14.3 μm and 10 μm; magnification × 700 and × 1000) and SEM micrographs with EDX spectra for pristine crab carapace (**e**–**f**) and the optimised CCM adsorbent (**g**–**h**) (Note: experimental conditions were impregnation ratio for KOH:carapace (g/g) 1:1; activation temperature 105 °C; activation time 150 min)

In order to assess if the KOH alkaline treatment had an effect on the porosity and textural structure of the crab carapace, SEM micrographs of raw material (Fig. 3a, b) and the optimised CCM adsorbent (Fig. 3c, d) were taken. SEM micrographs reveals some features of their irregular surface morphology and also some particle aggregation. From Fig. 3c, d, it is also obvious that the surface of the final CCM is rougher (compared to the raw material). The CCM had shown various cracks on the surface which are attributed to the deformation effect of the milling and activation. Cracks are randomly distributed over the CCM surface. The non-uniform distribution of cracks is due to the presence of the polymer. The cracks appeared brighter owing to the presence of calcite (highlighted on Fig. 3c, d).

Figure 3e–h further shows SEM-EDX spectra for the raw crab carapace and final CCM material. The EDX spectrum of the material after the optimisation with KOH shows the characteristic peaks for K, which confirms the presence of K along with other elements such as C, O, N, Mg and Ca, respectively, and shows the KOH has impacted on the process.

#### Surface and porosity characteristics

**Fig. 4:**
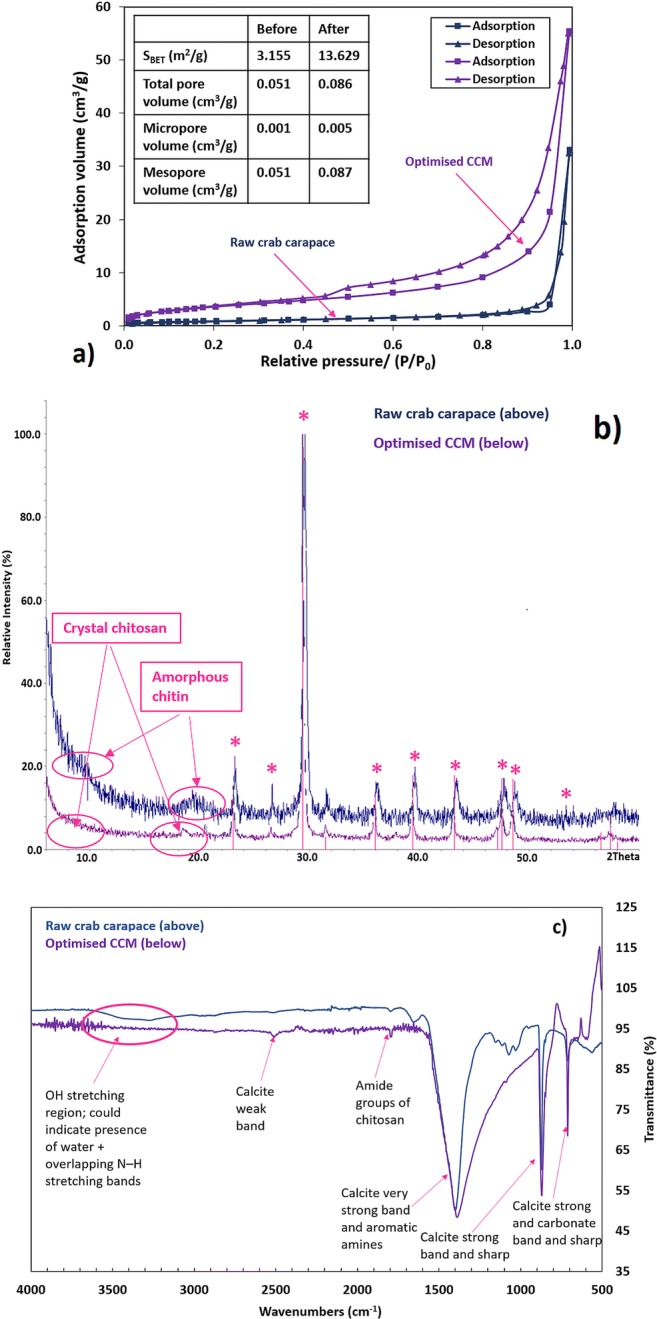
Nitrogen adsorption/desorption isotherms and BET data of raw crab carapace and the CCM adsorbent after optimisation (**a**), XRPD patterns of raw crab carapace and the optimised CCM adsorbent (Note: * reflections associated with calcite) (**b**) and FTIR spectra of raw crab carapace and the optimised CCM adsorbent (experimental conditions: impregnation ratio for KOH:carapace (g/g) 1:1; activation temperature 105 °C; activation time 150 min)

N_2_ adsorption/desorption isotherms are widely used to determine the surface area and porosity of adsorbents. The N_2_ isotherm data for the raw carapace material and CCM material are shown in Fig. 4a, which exhibits a type IV isotherm with a H4 hysteresis loop (IUPAC classification) at P/P_0_ = 0.995. This clearly indicates that both materials are mesoporous in nature. N_2_ adsorption in the region of P/P_0_ < 0.1 for CCM further indicates the coexistence of micropores. The *S*_BET_ surface area, total pore volume, micropore volume and mesopore volume of the two materials are summarised in Fig. 4a. The specific surface area for the crab carapace (before activation) was 3.155 m^2^/g, which increased to 13.629 m^2^/g post-activation. Likewise, total pore volume increased from 0.051 to 0.086 cm^3^/g.

### XRPD

The raw and activated materials were characterised by XRPD (Fig. 4b) to identify the phase(s) present in each sample. The normalised XRPD patterns show the presence of both an amorphous phase, which is associated with the organic component of the material, e.g. chitin/chitosan polymer and a crystalline phase, which was identified as calcite (Swanson et al. 1953; Zhou et al. 2016; Cai et al. 2019). The broad diffraction peaks observed at around 9.6° and 19° in the raw material (representing the amorphous chitin structure) are found to sharpen after activation, suggesting the material has crystallised during the activation process (Arulvel et al. 2017). These peaks were related to semi-crystal-I and semi-crystal-II in the chitosan structure (Arulvel et al. 2016).

### FTIR analysis

In order to explore differences between surface functionalities, FTIR spectra were recorded (Fig. 4c). Bands in the region 3600–3200 cm^−1^ correspond to O–H stretching vibration of carboxylic and hydroxyl groups, and water, which may overlap with several N–H stretching bands. The very strong asymmetric band at ~ 1400 cm^−1^ relates to the aromatic amines and CO_3_ in calcite. Characteristic absorption bands at 1800 and 1250 cm^−1^ are assignable to C=O stretching (amide I), N–H bending (amide II) and C–N stretching (amide III), especially characteristic of chitin/chitosan. The weak adsorption peak at 2500 cm^−1^ corresponded to bending vibrations of calcite. In both materials, peaks at 1150, 870 and 700 cm^−1^ were attributable to carbonate from the calcite in the crab carapace (Rashmi and Karthikeyan 2016; Samadi et al. 2018). The major differences between the two spectra are reflected in following: after the activation, the intensities of the characteristic peaks attributed to the vibrations of the amide group around 1800 cm^−1^ (ν_C=O_) decreased. In addition, the absorption peaks for calcite at 1150, 870 and 700 cm^−1^ increased.

### TGA analysis

**Fig. 5:**
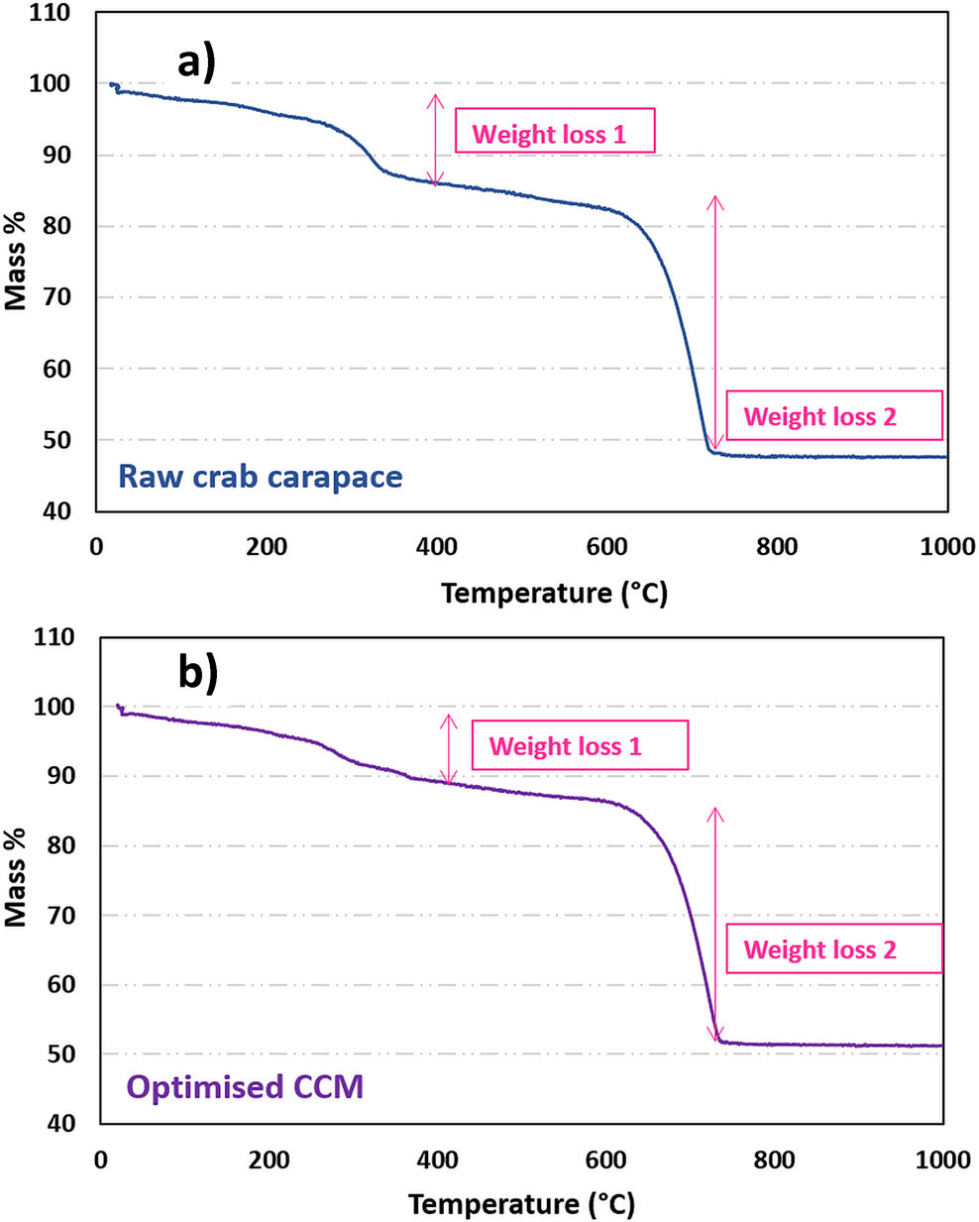
TGA curves of raw crab carapace (**a**) and optimised CCM (**b**) adsorbent in nitrogen atmosphere (Note: experimental conditions—impregnation ratio for KOH:carapace (g/g) 1:1; activation temperature 105 °C; activation time 150 min)

Thermal stability was investigated using thermogravimetric analysis (TGA) in a nitrogen atmosphere. As shown in Fig. 5a, b, thermal decomposition of the two samples occurred under slightly different thermal processes. Initial limited mass loss at temperatures below 150 °C was observed in both TGA curves—due to the vaporisation of adsorbed water. The first significant mass loss (11.3% for raw material and 9.3% for CCM), occurred at 266–343 °C and 216–378 °C, respectively. The second, larger weight loss (39.2% for raw material and 37.8% for CCM) was observed at temperatures between 600 and 740 °C and would be associated with the decarboxylation of the calcite.

## Discussion

### Modification and optimisation process

The effect of activation temperature on adsorbent efficiency has been noted (Abdolali et al. 2015). In general, adsorption performance is enhanced by increased activation temperatures—with thermal activation having greatest influence on surface area and porosity enhancement (Abdolali et al. 2015). Here, thermal treatment increased P removal to some extent, but a much greater P removal was found in materials which had undergone the alkali KOH treatment (Fig. 1a). Generally, KOH/NaOH can be used to hydrolyse protein present in an adsorbent, whilst strong acids (such as HCl) remove calcium salts (i.e. demineralisation) and can protonate unavailable functional groups, transforming them to carboxylic groups (Nadeem et al. 2008; Feng et al. 2009). Velazquez-Jimenez et al. (2013) further concluded that alkali treatment made adsorbent functional groups denser, and thermodynamically more stable, with a parallel increase in specific surface area.

If we consider the structure of crab carapace, it naturally contains approximately 20–29% chitin on a dry weight basis. Chitin, a long chain polymer comprising β-(1–4)-linked N-acetylglucosamine residues, is the second most abundant natural biopolymer on earth (Chu 2002). It can be converted to chitosan by deacetylation using a strong alkaline solution, such as KOH. Deacetylation of chitin to chitosan is represented by Fig. S4.

Chitosan is a highly versatile molecule, with many cited commercial applications in water treatment. Previous reports have demonstrated the effectiveness of chitosan in the uptake of phosphate as well as other oxyanions and metals (e.g. arsenate, arsenite, nitrate, strontium) (Jiang et al. 2013; Sowmya and Meenakshi 2013; Kumar and Jiang 2016; Liu et al. 2016; Rae et al. 2019). The binding capacity of chitosan to P is largely due to amine groups (–NH_2_) present on the chitosan chain, which can serve as coordination sites for many oxyanions (Fig. S4). Extent of P adsorption is also considered relative to the degree of deacetylation, the nature of the oxyanions present, and the solution pH (Chu 2002). In considering the P uptake capacity of the raw material tested here, alongside the three modified adsorbent materials prepared during the initial experiments (Fig. S1), we can conclude that relatively mild deacetylation (with 1 M KOH) had a high (the highest tested) influence on adsorption. This would highlight the importance of the chitin to chitosan conversion reaction on the periphery of the crab carapace.

Our results also indicate that IR (ratio of KOH: carapace (g/g) - 1:1) had the greatest impact on resultant removal efficiency. One possible explanation for this is that IR can correspond with increased porosity and chemically active surface development during activation. Presumably, activation with KOH caused an increase in surface cracking and/or the removal/displacement of impurities from partially blocked pores. Converting chitin to chitosan (in crab carapace) is a relatively slow chemical process, and it consumes significant amounts of reagent. Hence, increasing IR made the deacetylation process more efficient, aiding enhanced development of chitosan on both the exterior and interior surface of the CCM (Chu 2002). KOH also increases the pH_pzc_ at which the surface of the adsorbent is electroneutral (pH_pzc_ = 8.78), and it has been reported that the pH_pzc_ of similar calcium-chitin–based adsorbents are in the range of 9–11.4 (Tap Van et al. 2018). Theoretically, below this value, the surface of the material in solution is positively charged. This could then promote outer-sphere complexation/electrostatic attraction between the adsorbent and phosphate anions in solution even at neutral environmental condition. Increased activation time may also increase the extent of precursor-KOH reactions, thereby facilitating the development of pore structure, resulting in the formation of more chitosan. The lack of impact noted here due to activation temperature can be attributed to the high thermal stability of crab carapace (in the range 80–500 °C) which leads to the conclusion that the surface chemistry had greater influence on the P uptake compared with porosity (Mahmood et al. 2017). Overall, the degree of importance of the three independent variables studied here (in terms of maximising P adsorption capacity) were in the order of IR > activation time > activation temperature.

For the preparation of adsorbent material, high production yield and high removal efficiency are both desirable. High production yield helps reduce the cost of the adsorbent and high removal efficiency improves competitiveness versus other adsorbents within the commercial market. The price of an adsorbent, together with its successful commercial application, depends largely on four main factors: precursor cost, transportation cost, supply cost (chemicals and gases needed) and power consumption to produce (Selvaraju and Bakar 2017).

Referring to Table S2 and Eq. (S1), the calculated cost involved in the production of this CCM is estimated at 0.561 US$/kg (561 US$/ton) (Ahmed et al. 2016). According to current information, the cost of commercial adsorbents in the world market varies between ~ 800 and 5000 US$/ton (depending on the quality/type of adsorbent) (Selvaraju and Bakar 2017). The optimised activation method proposed here requires short processing times and low temperatures/heat energy, potentially three times less energy than that required for conventional thermochemical activation at higher temperature (500–1000 °C) (Pap et al. 2017). As such, this approach indicates that this material could be economically feasible, demonstrating the potential for this CCM as a scaled-up commercial product.

### Material characterisation

Structural changes on the CCM surface (Fig. 3a–d) due to alkali treatment suggest that processing not only rinses residual material left on the material, but it also dissolves some components from the raw material (i.e. proteins and fats). This in turn aids development of pores and increases porosity and the surface area of the adsorbent. This is confirmed by the BET analysis (Fig. 4a). Increased cracks and pores facilitates the diffusion of P into the interior of the CCM and provides a larger contact surface, i.e. more binding sites for P. Similar results have been obtained when other biomasses (i.e. combinations of tea waste, maple leaves and mandarin peel) were treated with 1.0 M NaOH (Abdolali et al. 2015). If we compare EDX spectra before and after activation, a new K peak is detected on the CCM surface, as might be expected. This indicates that during low-temperature activation, abundant K ions become bound to the CCM (Šoštarić et al. 2018). The presence of all mono- and divalent ions on the CCM surface will assist in the formation of new Ca-P, Mg-P and K-P crystals. This precipitation phenomena is also generally favoured at alkaline pH. The prepared adsorbent here had a relatively high pH_pzc_ (8.78), which would induce an increase in the aqueous solution pH; consequently, this may then contribute to P precipitation with calcium, magnesium and potassium, i.e. as various phosphates (Haddad et al. 2018).

The main phase identified here in all samples was calcite (International Centre for Diffraction Data; PDF 5-586). The peak breadths of the raw material were wider than in the CCM, indicating a difference in the crystallite size before and after treatment (before treatment, crystallites were smaller). A broad bump at ~ 19° (see Fig. 4b) was also present in the raw material, which may indicate the presence of biopolymer amorphous components/ biopolymers (i.e. the semi crystalline structure of chitin). Finally, the diffraction peak of the (104) plane is the most intense peak for calcite in CCM, indicating that CaCO_3_ in the crab carapace is in crystallised calcite form along the biopolymer chains (Cai et al. 2019).

In term of the FTIR data (Fig. 4c), no significant differences between the two spectra before and after modification were seen (i.e. the positions of the main absorption peaks did not changed drastically). The most obvious slight change happened within the low wavenumber region (1200–500 cm^−1^). The presence of the amines generally acts as a complexation sites for the P to attaches on the surface of CCM. The presence of the amine groups and calcite on the CCM is the major factor for the interaction with P ions. Whilst modification of the crab carapace did not change the primary functional groups significantly (on the surface), adsorption capacity did improve to some extent. Again, this may have been primarily caused by chitin deacetylation, crystallisation and observed increases in specific surface area and porosity.

TGA analysis (Fig. 5a, b) showed that raw and activated material exhibited medium thermal stability. Results indicated that samples lost ~ 50% by mass, likely due to (i) removal of physically bonded water and (ii) decomposition of pure CaCO_3_ and/or decarboxylation at higher temperature (in range 650–850 °C). The expected weight loss for pure CaCO_3_ alone would be 43.96% and would follow this reaction:

6 $$ {\mathrm{CaCO}}_3\to \mathrm{CaO}+{\mathrm{CO}}_2 $$

When comparing material before/after activation, the thermal stability of the CCM was higher after KOH treatment (total weight loss reduced from 50.5 to 47.1%). This extra stability could be due to the reaction between KOH and the CCM surface (less decomposition of the organic components).

## Conclusion

A chitosan-calcium–rich adsorbent was prepared from raw waste crab carapace with the aid of a BBD optimised approach. The optimum conditions found were KOH:carapace (g/g) ratio 1:1, activation temperature 105 °C and activation time 150 min. The degree of importance of these three independent variables on preparation of the final CCM (to maximise P adsorption capacity) was KOH:carapace ratio > activation time ≈ activation temperature. The approximate calculated cost involved in production of this CCM was estimated at 0.561 US$/kg (561 US$/ton). This may indicate that it could be economically feasible to produce this material at a commercial scale. Characterisation (using SEM/EDX, XRPD, FTIR, TGA and BET) suggested that KOH acted as a powerful activation agent. Results suggest that during synthesis, chitin was deacetylated to chitosan, and multitude amine groups (–NH_2_) on the chitosan chain would serve as coordination sites for P. The final CCM also possessed higher crystallisation, thermal stability, a larger surface area and higher porosity. Results indicated that low-temperature activation of crab carapace (*Cancer pagurus*) to create a P adsorbent has promise as a valorisation conversion method for this food-processing waste. Future research will focus on understanding P adsorption mechanisms onto this CCM—explored through kinetic, dynamic, isotherm and thermodynamic studies and instrumental characterisation.

## Electronic supplementary material

ESM 1 (DOCX 236 kb)
