# Supplementary material for: Synthesis optimisation and characterisation of chitosan-calcite adsorbent from fishery-food waste for phosphorus removal
Source: Environ Sci Pollut Res Int. 2020 Jan 11;27(9):9790–802. doi: 10.1007/s11356-019-07570-0 (PMC7089908; doi:10.1007/s11356-019-07570-0)
Supplement: Supplementary file 1 — (DOCX 236 kb) [file 11356_2019_7570_MOESM1_ESM.docx]

**Supplementary material**

**Synthesis optimisation and characterisation of chitosan-calcite adsorbent from fishery-food waste for phosphorus removal**

**Sabolc Pap^a,b,^**^^[[1]](#footnote-1)^^**, Caroline Kirk^c^, Barbara Bremner^a^, Maja Turk Sekulic^b^, Stuart W. Gibb^a^, Snezana Maletic^d^, Mark A. Taggart^a^**

*^a^ Environmental Research Institute, University of the Highlands and Islands, Thurso, Caithness, Scotland, KW14 7JD, UK*

*^b^ University of Novi Sad, Faculty of Technical Sciences, Department of Environmental Engineering and Occupational Safety and Health, Novi Sad, Serbia*

*^c^ School of Chemistry, University of Edinburgh, David Brewster Rd, Edinburgh, EH9 3FJ, UK*

*^d^ University of Novi Sad, Faculty of Science, Department of Chemistry, Biochemistry and Environmental Protection, Trg Dositeja Obradovića 3, Novi Sad, Serbia*


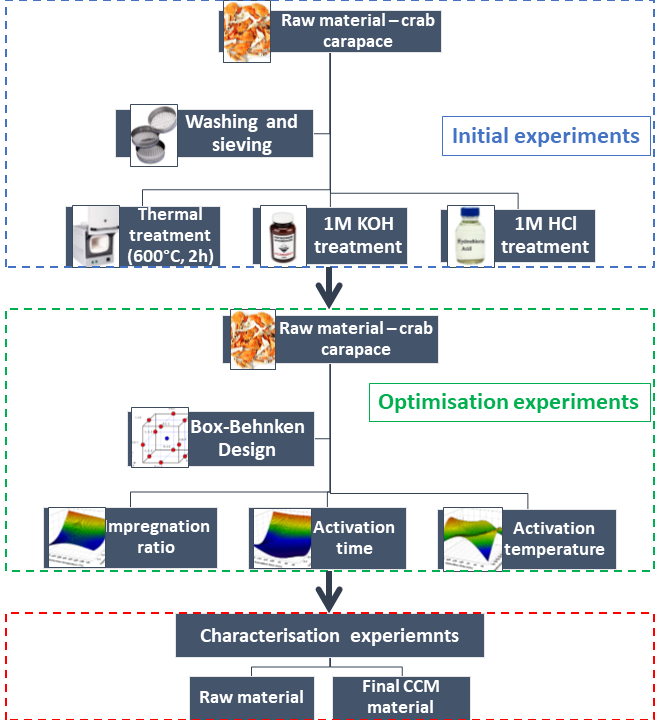


**Fig. S1.** Schematic representation of the experiments - illustration of the sequential experimental process

**Fig. S2.** Correlation of actual and predicted values of response from BBD method


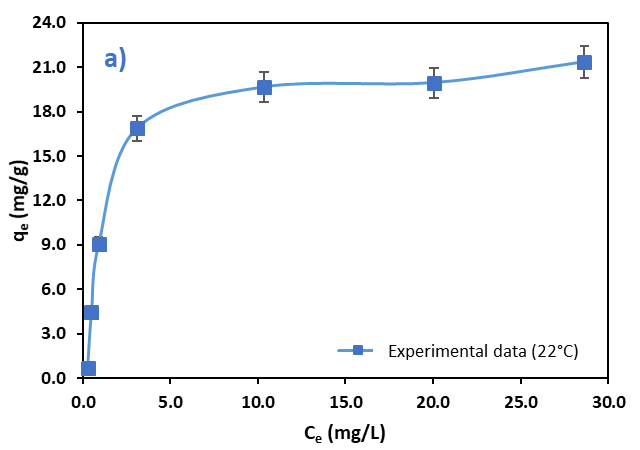

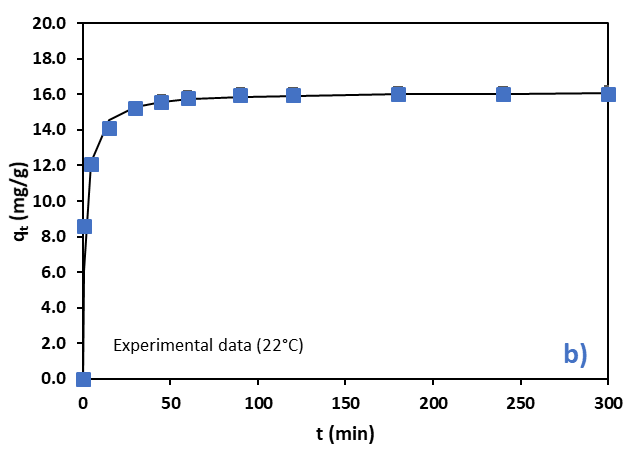


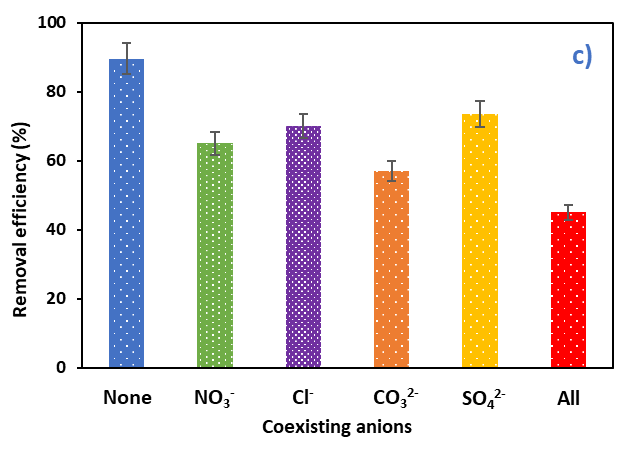


**Fig. S3.** Adsorption isotherm (a), removal rate (b) and selectivity (b) of P onto CCM optimised adsorbent


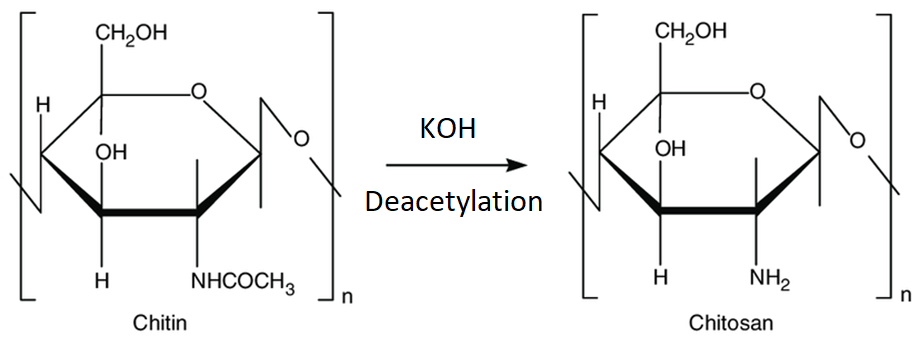


**Fig. S4.** Representation of the conversion of chitin to chitosan by deacetylation in the presence of KOH.

**Table S1**

Model validation parameters

| IR (g/g) | Activation temperature (°C) | Activation time (min) | Removal efficiency (%) | |
| --- | --- | --- | --- | --- |
|  |  |  | Predicted | Experimental |
| 1 | 105 | 150 | 75.89 | 78.18 |

The production cost of this CCM (US$/ton) in Scotland could be calculated as Eq. (S1):

$$CCM=CRM+CT+CCRM+CSR+DRM+CH+COA+COW+DAC (S1)$$

**Table S2.** Estimated production cost of CCM (US$/kg) [1]

| Components | Description | US$/kg |
| --- | --- | --- |
| Cost of raw material (CRM) | The raw material is locally and abundantly available. | 0.0 |
| Cost of transport (CT) | Difficult to predict, 10% surcharge should be added to the overall cost | 0.05 |
| Cost of cleaning raw material (CCRM) | The raw material was washed with tap water. The cost of water usage (the price of tap water per liter × water consumption for 1 kg) | 0.001 |
| Cost of size reduction (CSR) | The crab carapace was crushed in a mechanical mill. The electricity consumption for 1 kg × cost of 1 unit = 0.1 × 0.2 | 0.02 |
| Cost of drying raw material (DRM) | Dried at room temperature. | 0.0 |
| Cost of hydroxide (CH) | Potassium hydroxide | 0.12 [2] |
| Cost of the optimised activation (COA) | Cost of heating = hours × units × per unit cost = 2.5 × 0.3 × 0.2 | 0.15 |
| Cost of adsorbent washing (COW) | The sorbent was washed with ultra-pure water. | 0.10 |
| Cost of drying the adsorbent (DAC) | Hours × units × per unit cost = 2 × 0.3 × 0.2 | 0.12 |
| Overall cost | | 0.561 |

**References**

[1] G. Selvaraju, N.K.A. Bakar, Production of a new industrially viable green-activated carbon from Artocarpus integer fruit processing waste and evaluation of its chemical, morphological and adsorption properties, J. Clean. Prod. 141 (2017) 989–999. doi:10.1016/j.jclepro.2016.09.056.

[2] R.K. Liew, E. Azwar, P.N.Y. Yek, X.Y. Lim, C.K. Cheng, J.H. Ng, A. Jusoh, W.H. Lam, M.D. Ibrahim, N.L. Ma, S.S. Lam, Microwave pyrolysis with KOH/NaOH mixture activation: A new approach to produce micro-mesoporous activated carbon for textile dye adsorption, Bioresour. Technol. 266 (2018) 1–10. doi:10.1016/j.biortech.2018.06.051.

1. Corresponding author at: Environmental Research Institute, University of the Highlands and Islands, Scotland, KW14 7JD, UK

   *E-mail address:* [szabolcs.pap@uhi.ac.uk](mailto:szabolcs.pap@uhi.ac.uk); [sabolcpap@uns.ac.rs](mailto:sabolcpap@uns.ac.rs)

   Telephone: 01847 889676; Fax: 01847 890014

   ORCID: 0000-0001-7395-1913;

   Notes: The authors declare no competing financial interest. [↑](#footnote-ref-1)
